# Single-cell profiling demonstrates the combined effect of wheeze phenotype and infant viral infection on airway epithelial development

**DOI:** 10.1126/sciadv.adr9995

**Published:** 2025-05-23

**Authors:** Sergejs Berdnikovs, Dawn C. Newcomb, Nana-Fatima Haruna, Kaitlin E. McKernan, Shelby N. Kuehnle, Tebeb Gebretsadik, Christopher McKennan, Siyuan Ma, Jacqueline-Yvonne Cephus, Christian Rosas-Salazar, Larry J. Anderson, James E. Gern, Tina Hartert

**Affiliations:** ^1^ Division of Allergy and Immunology, Department of Medicine, Northwestern University Feinberg School of Medicine, Chicago, IL, USA.; ^2^ Department of Medicine, Vanderbilt University Medical Center, Nashville, TN, USA.; ^3^ Department of Biostatistics, Vanderbilt University Medical Center, Nashville, TN, USA.; ^4^ Department of Statistics, University of Pittsburgh, Pittsburgh, PA, USA.; ^5^ Department of Pediatrics, Vanderbilt University Medical Center, Nashville, TN, USA.; ^6^ Department of Pediatrics, Emory University School of Medicine and Children’s Healthcare of Atlanta, Atlanta, GA, USA.; ^7^ Department of Pediatrics, University of Wisconsin School of Medicine and Public Health-Madison, Madison WI, USA.

## Abstract

The development of the airway epithelium in asthma is unclear. We characterized nasal airway epithelial cell (NAEC) developmental phenotypes from children aged 2 to 3 years in an a priori designed nested birth cohort from four mutually exclusive groups of wheezers/nonwheezers and respiratory syncytial virus (RSV)–infected/uninfected in the first year of life. NAECs were differentiated, followed by single-cell RNA sequencing analysis and in vitro RSV infection. Gene expression of NAECs from children with a wheeze phenotype indicated abnormal differentiation and basal cell activation of developmental pathways, plasticity in precursor differentiation, delayed onset of maturation, increased diversity of RSV receptors, and blunted antiviral immune responses to in vitro RSV infection. The most marked changes in differentiation were observed in NAECs from children with both wheeze and RSV in the first year of life. Together, this suggests that airway epithelium in children with wheeze is developmentally reprogrammed and characterized by increased barrier permeability, decreased antiviral response, and altered RSV receptor expression.

## Teaser

Airway epithelial cells from children with wheeze are characterized by altered development and susceptibility to RSV infection.

## INTRODUCTION

Childhood asthma is in part an airway epithelial developmental disorder, and its origin and clinical manifestations are tightly linked with altered airway epithelial cell (AEC) physical, metabolic, and functional barrier properties. The airway epithelium consists of multiple specialized cell subsets forming a functional barrier against the external environment. Many of the risk genes for asthma are expressed in the airway epithelium, particularly allergy and epithelial barrier function genes, which support the importance of the airway epithelium in asthma development (*1*, *2*). AEC barriers in childhood are shaped and regulated by active ongoing developmental programs, with morphogenesis of lung epithelial cells and barrier function continuing throughout normal postnatal development (*3*–*7*). AEC differentiation occurs largely in the first year of life but continues until ~2 years of age. Asthma results from host and environment interactions and environmental asthma risk factors such as pollution, tobacco smoke, and respiratory viruses interact directly with the developing airway epithelium (*8*). Since AEC development continues after birth, early-life mucosal environmental exposures have the unique opportunity to alter the course of AEC differentiation and contribute to asthma development.

Respiratory syncytial virus (RSV) is an airway mucosal pathogen, which directly infects the airway epithelium and is one of the most consistently identified asthma risk factors with a high population-attributable fraction (*9*, *10*). Respiratory viruses represent a unique early-life exposure, as they integrate into the cell to replicate, in contrast to inhalant and irritant exposures. RSV serves as an ideal model to understand the impact of early-life environmental exposures on airway epithelial development in both in vivo and in vitro studies, as about half of infants are infected with RSV in the first year of life, providing comparator groups of infected and uninfected infants (*10*, *11*). Airway epithelial changes have been demonstrated in children before the onset of asthma, suggesting that airway epithelial developmental changes occur early in asthma pathogenesis. However, single-cell profiling of wheeze, which typically precedes asthma, and the impact of early-life mucosal respiratory viruses such as RSV on epithelial developmental phenotype have never been studied (*12*).

The objective of this study was to test whether (i) there are unique developmental characteristics of the early-life airway epithelium that characterize childhood wheeze, including the manifestation of wheeze developmental phenotype under air-liquid interface (ALI) differentiation culture conditions, and (ii) early-life asthma risk factors, using natural infant RSV infection as a model, affect AEC developmental programming and AEC differentiation in culture. As the collection of infant and child bronchial epithelium is highly invasive and impractical in population-based studies, we used the nasal airway epithelium to characterize developmental phenotypes in health and disease. The nasal transcriptome has been demonstrated to be an excellent and well-accepted proxy of expression changes in the lung airway transcriptome in asthma, as well as in distinguishing phenotypes of asthma (*13*–*15*). We used nasal AECs (NAECs) cultured under ALI differentiation conditions, in combination with single-cell RNA sequencing (scRNA-seq) and in vitro infection with RSV to investigate AEC developmental phenotypes at the age of 2 to 3 years that characterize children with wheeze and to determine whether an early-life environmental asthma risk factor, RSV, is associated with altered NAEC development.

## RESULTS

### Selection of study population based on wheeze and RSV exposures in infancy

**Fig. 1.**
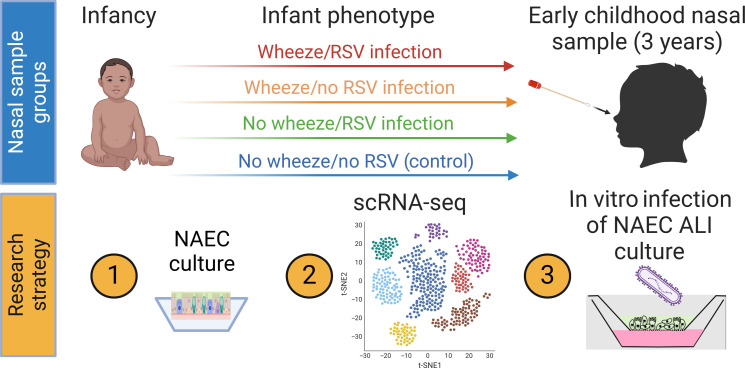
Graphical outline of research study workflow. Figure was created in BioRender (T. Hartert, 2025; https://BioRender.com/r41p718).

To determine whether wheeze and early-life (before 1 year of age) RSV infection are associated with altered AEC development, we collected NAECs from children aged 2 to 3 years, which represents the end point of postnatal differentiation trajectory, in the Infant Susceptibility to Pulmonary Infections and Asthma Following RSV Exposure study (INSPIRE) birth cohort. INSPIRE is a population-based birth cohort of healthy, term, and predominantly non–low birth weight infants. The subgroup for this study was selected from an a priori designed nested cohort of 100 participants using a random number generator from four predefined groups based on their history of wheezing and confirmed presence/absence of RSV infection during the first year of life. The demographic characteristics of the nine participants in this substudy to the full cohort are shown in table S1. NAECs at age 2 to 3 years were collected from these four a priori groups: control (no wheeze/no infant RSV infection), RSV in the first year of life (no wheeze/infant RSV infection), wheeze starting in the first year of life (wheeze/no infant RSV), and wheeze and RSV in the first year of life (wheeze/RSV) (fig. S1). Wheeze was tracked annually using a validated questionnaire, and RSV infection by age 1 year was defined with a combination of passive and active surveillance, with viral identification through molecular and serological testing to identify RSV infection (*16*, *17*). Two to three participants from each of these four mutually exclusive groups were randomly selected for scRNA-seq, and five to six different samples were selected from each group for in vitro experiments. Table S2 includes data on the participants included in the study, stratified by a priori study group based on 1-year wheeze and RSV infection. Samples were not chosen to balance by sex, given the limited sample size. All male samples were selected unless there were no available samples. Figure 1 illustrates the use of NAECs that were collected and cultured at ALI to allow for differentiation from the four a priori defined groups and single-cell analysis. In additional experiments, transepithelial electrical resistance (TEER) was measured during NAEC differentiation, and NAECs from the four a priori defined groups were infected with a clinically relevant strain of RSV, RSV 01-2/20, and mock to measure RSV infectivity.

### scRNA-seq analysis of differentiated NAECs from 2 to 3 years old identifies epithelial subset composition by wheeze and RSV phenotypes

**Fig. 2.**
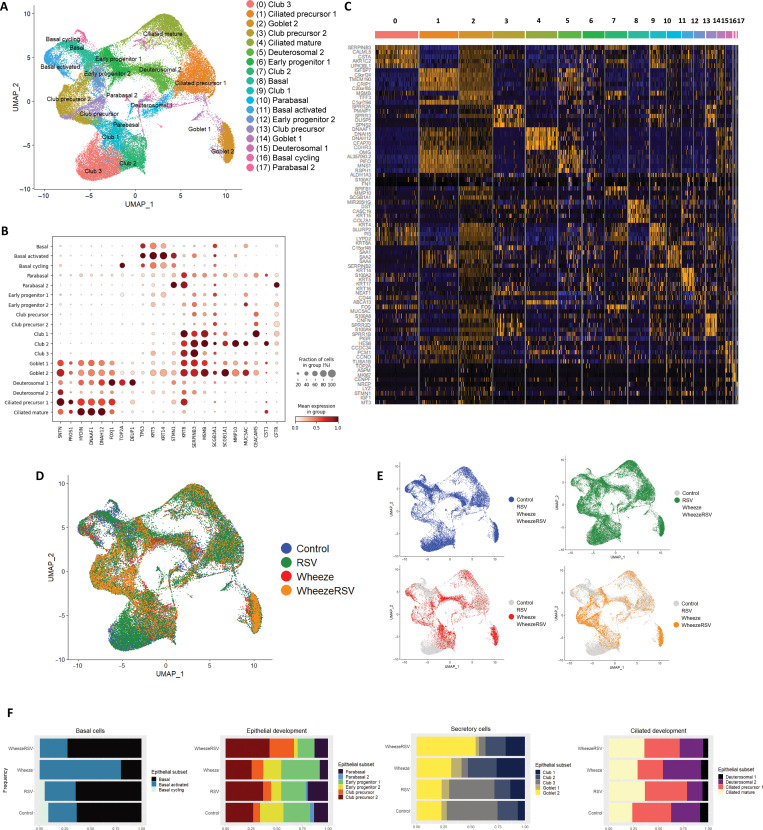
Epithelial subset composition of the developing NAECs (2 to 3 years old) in ALI culture. (**A**) An integrated object from all four study groups showing identified epithelial cell subsets. UMAP, Uniform Manifold Approximation and Projection. (**B**) Dot plot showing conventional markers used to identify epithelial cell subsets. (**C**) Top markers for each of the 17 clusters identified by differential gene expression analysis. Cluster numbers on top correspond to cluster numbering in (A). Low expression is shown in blue and high expression is shown in orange. (**D**) An integrated object from all four study groups [wheeze/RSV, wheeze/no RSV, no wheeze/RSV, no wheeze/no RSV (control)] showing cell subset differences by group. (**E**) An integrated object from all four study groups highlighting cells individually by group. (**F**) Proportional representation of cells per cluster by study group. From left to right: basal cell clusters, progenitor and precursor clusters, secretory clusters, and ciliated epithelial clusters. Study groups: wheeze/RSV in infancy, wheeze (wheeze/no RSV), RSV (no wheeze/RSV in infancy), and control (no wheeze and no RSV in infancy).

First, we determined epithelial subset composition of the NAECs collected from children between ages 2 and 3 years and cultured in ALI. We performed an integration scRNA-seq analysis of all epithelial samples and determined cell subset composition based on known markers of epithelial differentiation as published by Vieira Braga *et al.* (*18*). We identified 17 different epithelial clusters across the four study groups, including basal, progenitor, and mucociliary subsets (Fig. 2, A to C). Analysis of top marker genes differentially expressed across the 17 clusters further confirmed functional identity of these subsets (Fig. 2C). Despite shared subset identity, several subsets of epithelial cells clustered separately on the basis of group origin (i.e., three distinct clusters of club cells, two clusters of goblet cells, and various precursors), likely driven by functional or developmental differences in the AEC phenotypes (Fig. 2, A and B). Next, we compared subset composition of epithelial cells across the four prespecified groups: (i) no wheeze/no RSV infection in infancy (control, −/−), (ii) no wheeze/RSV infection in infancy (−/+), (iii) wheeze/no RSV infection in infancy (+/−), and (iv) wheeze/RSV infection in infancy (+/+). Figure 2D shows cell integration from all four study group samples, summarizing cell subset differences by group. There were appreciable differences in basal, precursor, and club cells across different groups, especially in wheeze groups, while the RSV no wheeze group appeared more similar in epithelial composition to the control group (Fig. 2E). Next, we specifically measured differences in proportion of each epithelial subset across the four study groups, broadly grouping subsets as basal cell clusters (basal, basal activated, and basal cycling), epithelial development clusters (parabasal, early progenitor, and club precursor subsets), secretory cells (club and goblet cells), and ciliated development (deuterosomal and ciliated precursor and mature epithelial cells) (Fig. 2F). There was a relative decrease in basal cycling cells in both wheeze groups compared to control and no wheeze/RSV groups. We also found notable changes in composition of goblet and club epithelial cell clusters that were specific to wheeze groups, while the epithelial cells from the RSV only and control groups were similar (Fig. 2F). In summary, ALI culture represented well the differentiation pattern of the epithelium, enabling comparative study of epithelial clusters across the four study groups. Together, cluster comparisons across study groups suggested a specific developmental defect common to the wheeze phenotype, while changes related to RSV infection were not as pronounced as in the wheeze subgroups.

### Characterization of normal versus wheeze airway epithelial phenotypes and differentiation trajectories in ALI

**Fig. 3.**
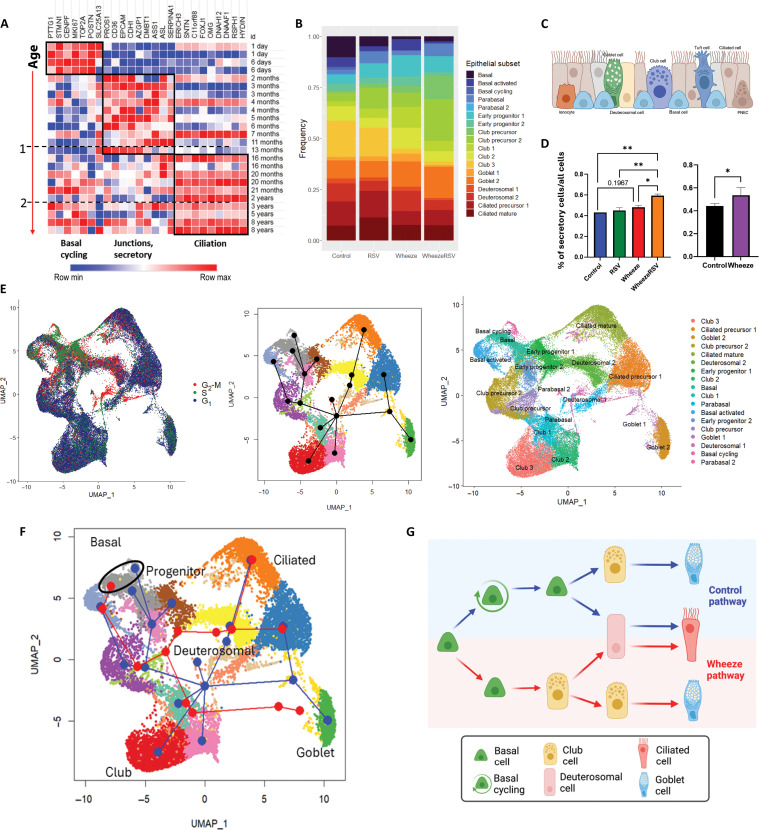
NAECs from wheeze study groups show evidence of developmental reprogramming and an increased expansion of epithelial precursor and secretory phenotypes. (**A**) LungMAP data mining of human lung tissue development suggests specific epithelial differentiation trajectory in early life. (**B**) NAECs from wheeze study groups show trend of relative expansion of epithelial precursor and secretory subsets. (**C**) A diagram showing heterogeneous composition of mature stratified respiratory epithelium. (**D**) Epithelium from wheeze group samples showed a proportional increase in secretory (club precursor, club, and goblet subsets combined) cells relative to cells from all populations. Left: Individual samples across four groups quantified. Right: Samples combined by wheeze versus nonwheeze groups. *P < 0.05, **P < 0.01 by ANOVA (graph on the left) and t-test (graph on the right). (**E**) Cell cycle analysis confirms early identity proliferative capacity of basal and progenitor epithelial cell subsets. Cells from all groups are shown in the integrated object. (**F**) Unsupervised slingshot developmental trajectory inference reveals altered developmental trajectory in wheezers (red lines) compared to nonwheeze controls (blue lines). Black circle denotes basal cell populations as the starting point for developmental trajectories. (**G**) Interpretation of developmental trajectories in control and wheeze study groups. (G) was created in BioRender (T. Hartert, 2025; https://BioRender.com/r41p718).

We next sought to determine whether there are specific differences in epithelium from children with a history of wheeze that distinguish it from healthy (control) epithelium. Our data mining of human birth and early-life lung gene expression from a publicly available resource (the LungMAP database) demonstrates that epithelial postnatal development follows a specific trajectory, with predominant basal gene expression at birth, followed by increasing expression of cell junction and secretory signatures during the first year of life, and a signature of ciliation increasing after the first year of life (Fig. 3A). ALI culture conditions allow for in vitro study of a basal–to–specialized epithelial differentiation process, providing the opportunity to compare differentiation trajectories in control versus wheeze AEC phenotypes. Relative proportions of all epithelial subsets across our four study groups suggested a changing composition of epithelial barrier in the RSV-only group (no wheeze/RSV) relative to controls, but these changes were even more pronounced in wheeze groups (Fig. 3B). The mature epithelial barrier is known to be highly heterogeneous but functionally balanced with multiple subsets with specialized developmental, structural, secretory, sensory, and defense roles in the conducting airways (Fig. 3C). We found a trend of increase in developing and secretory epithelial subsets in wheeze groups, especially in epithelial cells derived from children who had both RSV and wheeze in the first year of life (Fig. 3D). Overall, combined samples from both wheeze groups showed increased secretory subsets (club precursors, club, and goblet cells) compared to all nonwheeze group samples (Fig. 3D). Cell cycle analysis confirmed an early identity of progenitor and precursor populations (expressing markers of G_2_-M and S cell cycle phases) with increased proliferation capacity (Fig. 3E). Slingshot reconstruction of differentiation trajectories of the epithelium revealed differences in the development of epithelial cells from the wheeze subgroups relative to control. This suggests that wheezing illnesses induce an alternative precursor differentiation event, and this distinct developmental pathway likely results in alternate mature subsets with functional differences (Fig. 3F). In summary, despite common origins in basal cells, airway epithelium from children with wheeze is characterized by differentiation along an altered trajectory with early basal activation of developmental pathways, plasticity in precursor proliferation and differentiation, and the delayed onset of maturation (summarized in Fig. 3G).

### Developmental programming and activation of basal cells in airway epithelium from children with wheeze and RSV infection in the first year of life

**Fig. 4.**
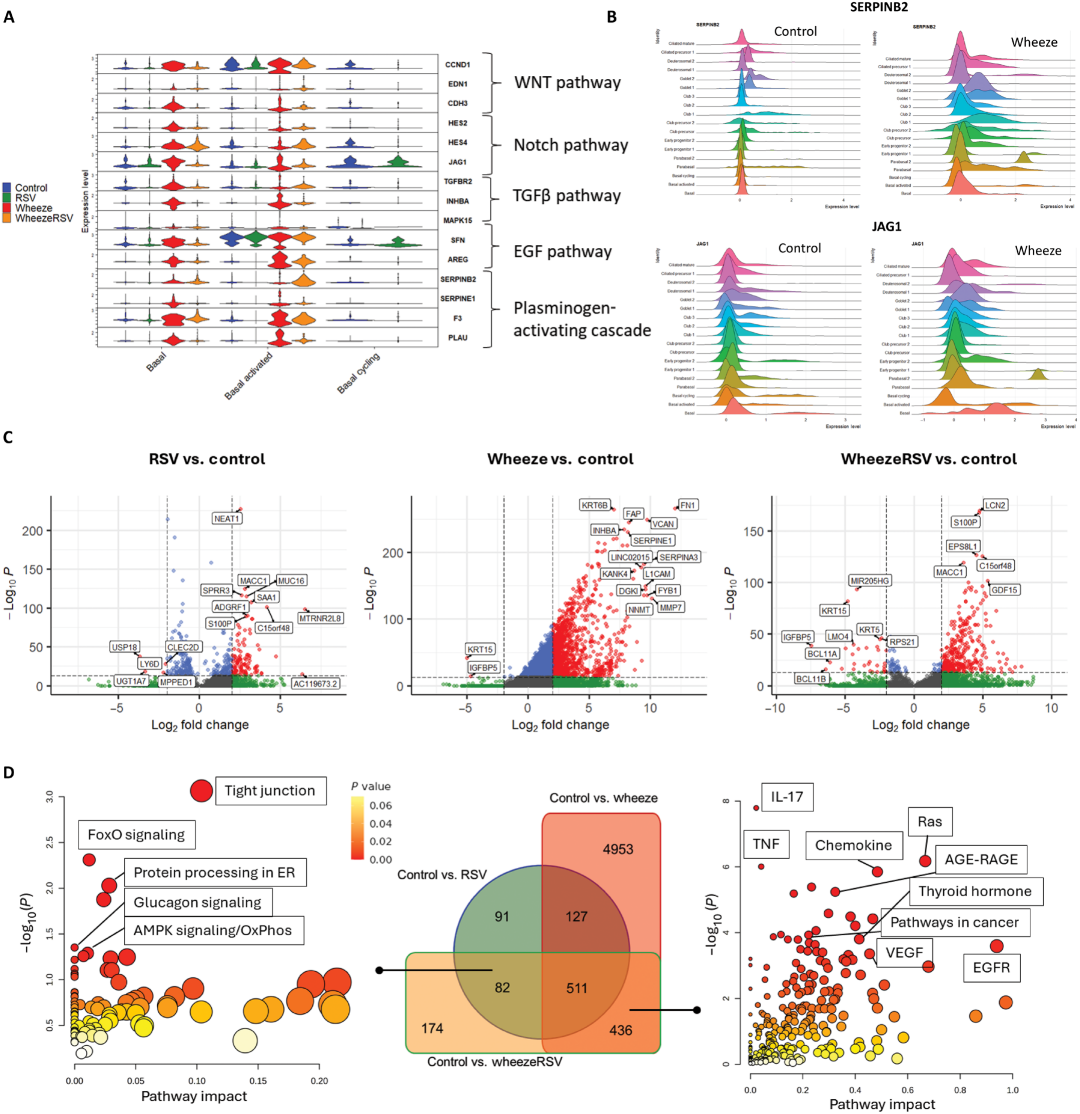
NAECs from wheeze study groups show early activity of developmental pathways and abnormal activation of basal cells. (**A**) Expression of markers of developmental pathways (WNT, Notch, TGF, EGF, and tissue plasminogen system) by cell type and study group. (**B**) Expression of PAI-2 (SERPINB2) and JAG1 (Notch/Jagged) is increased in wheeze NAECs. (**C**) Differentially expressed gene (DEG) analysis of basal cell subset comparing RSV and wheeze study groups to controls. False discovery rate–adjusted *P* values are used. (**D**) Pathway analysis of up-regulated basal cell DEGs common to control versus RSV and “control versus wheeze/RSV” (left) and up-regulated basal cell DEGs common to control versus wheeze and control versus wheeze/RSV (right). OxPhos, oxidative phosphorylation.

Since basal cells represent a starting point in epithelial differentiation, we next examined whether gene expression and biological processes in NAECs collected at age 2 to 3 years from infants with RSV infection in the first year of life or wheeze differ from controls (no wheeze/no first-year RSV infection), specifically in the basal cell subsets. First, we found increased expression of the developmental pathway [WNT, Notch, epidermal growth factor (EGF), transforming growth factor (TGF), and tissue plasminogen] markers specifically in the two wheeze groups (wheeze/no RSV and wheeze/RSV) relative to controls (no wheeze/no RSV), while these markers were not different between RSV-only and control groups (Fig. 4A). These markers were increased specifically in basal and basal-activated subsets, while basal cycling cells were lacking in wheeze groups. For example, expression of PAI-2 (*SERPINB2*) and *JAG1* (Notch/Jagged) was increased specifically in wheeze NAECs (Fig. 4B).

Next, we determined differentially expressed genes (DEGs) between each of our study groups relative to the control group (Fig. 4C). Wheeze and wheeze/RSV groups had the highest number of up-regulated genes relative to controls, while the no wheeze/RSV group had the lowest number of DEGs compared to controls (no wheeze/no RSV). Top up-regulated genes in NAECs from infants with first-year RSV infection included nuclear-enriched abundant transcript 1 (*NEAT1*), Mesenchymal-to-epithelial transition transcriptional regulator *MACC1*, mucin *MUC16*, and small proline-rich protein 3 (*SPRR3*). Top genes in NAECs from children with wheeze included fibronectin (*FN1*) and keratin 6B (*KRT6B*), while the wheeze/RSV group highly expressed lipocalin-2 (*LCN2*) and S100 calcium binding protein P (*S100P*). Keratin *KRT15* and insulin-like growth factor (IGF) binding protein 5 (*IGFBP5*) were commonly down-regulated in wheeze groups with and without infant RSV infection (Fig. 4C), thus potentially representing markers of wheeze phenotype. Biologically, down-regulation of KRT15 is associated with loss of differentiation of basal epithelium and promotion of epithelial-mesenchymal transition.

Next, to understand specific differences between wheeze and RSV basal cell activation, we performed a pathway analysis on up-regulated basal cell DEGs common to “control versus RSV” and “control versus wheeze/RSV” comparisons (thus representing RSV-only response) and up-regulated basal cell DEGs common to “control versus wheeze” and control versus wheeze/RSV comparisons (representing wheeze-specific biology) (Fig. 4D). We found that RSV basal activation is associated with pathways that include tight junction regulation, FoxO signaling pathway, protein processing in endoplasmic reticulum, glucagon signaling, adenosine 5′-monophosphate kinase (AMPK) signaling, and oxidative phosphorylation. Wheeze phenotype is characterized by basal cell activation and up-regulation of immune signaling pathways [interleukin-17 (IL-17), tumor necrosis factor (TNF), and chemokines], RAS advanced glycation end products (AGE) and their receptor, RAGE, thyroid signaling, and growth factor/remodeling pathways [vascular endothelial growth factor (VEGF), EGF, and pathways in cancer (WNT and Notch/Jagged)], among others in our dataset. Together, these findings show that the wheeze phenotype is associated with immune activation, remodeling, and abnormal developmental activity of basal cells, which likely sets wheeze NAECs on an aberrant differentiation trajectory, whereas infant RSV infection alone is associated with a more subtle impact on epithelial cellular processes that would lead to disruption of barrier development.

### Biological processes associated with extracellular matrix deposition and immune activation in NAECs by wheeze phenotype

Next, we performed an enrichment analysis to determine which biological processes and transcriptional programs are associated with aberrant development of NAECs from children with wheeze. Figure S2A shows biological enrichment analysis of genes expressed in different developmental clusters comparing control and wheeze-only groups. This analysis showed increased gene activity associated with remodeling of the extracellular matrix in basal cells from the wheeze group, consistent with the relative increase in basal-activated cells noted above. Notably, we also found evidence for sustained immune activation of epithelial cells from children with wheeze, specifically in parabasal, early progenitor, and goblet cells, evidenced by expression of chemokines, cytokines, and antigen presentation genes. These processes were not prominent in the NAECs from control children, which only showed processes consistent with normal epithelial development, keratinization, and junction formation (fig. S2A). Transcription factor inference analysis confirmed these functional differences, suggesting early activation of BRCA1 (Breast Cancer gene 1), Myc, and TGFβ pathway (SMADs) in children with wheeze and sustained progenitor transcription programs (JUN and JUND/B) (fig. S2B). We specifically noted increased expression of a panel of gene markers associated with remodeling (*SERPINB2*, *THBS1*, *TGFBI*, *TNC*, *COL1A1*, *VCAN*, *FN1*, *SPP1*, *TAGLN*, and *POSTN*) in airway epithelium of children with wheeze (fig. S2C). Notably, NAECs from children with wheeze expressed *KRT13* (marker of hillock cells) in early progenitor and club precursor subsets along with *CXCL8* (immune) and *CFB* (complement) markers, while remodeling markers such as *FN1* (fibronectin) noted above were expressed in basal and early progenitor subsets (fig. S2D). In summary, these results show that the wheeze epithelial developmental phenotype is characterized by aberrant activation of basal and club precursor cells and persistent activation of remodeling, complement, and immune pathways during development.

### Diversity of RSV receptors, expression of antiviral response genes, barrier permeability, and susceptibility to in vitro RSV infection in NAECs from wheeze and no wheeze subgroups

**Fig. 5.**
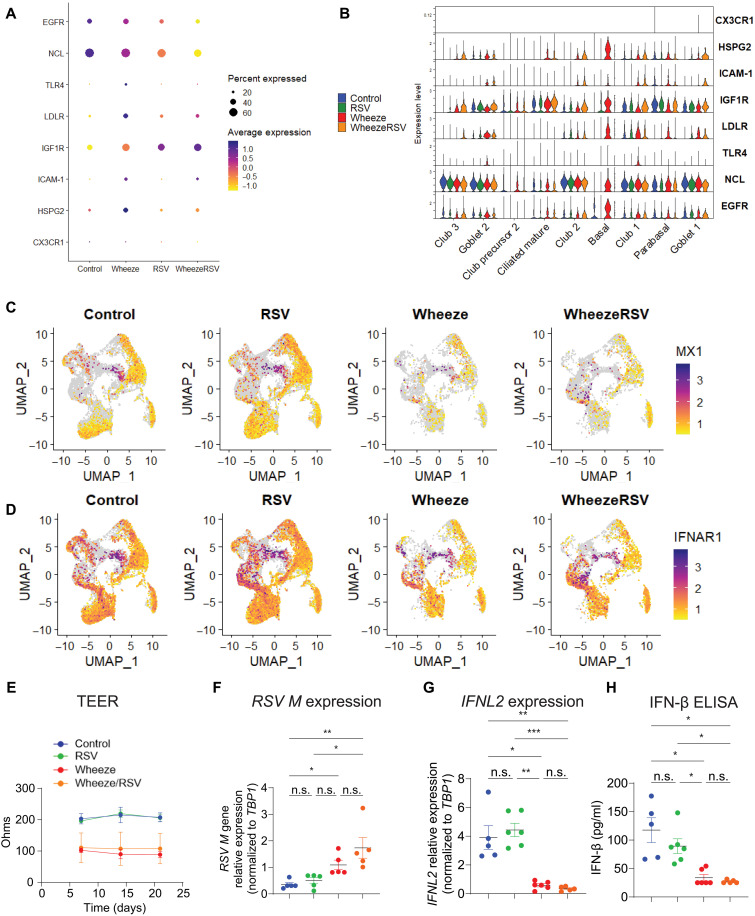
NAECs from wheeze study groups show increased diversity of RSV receptors, decreased expression of host antiviral response genes, increased barrier permeability, and increased susceptibility to RSV infection in vitro. (**A** and **B**) NAECs from wheeze groups show increased diversity of RSV receptors. TLR4, Toll-like receptor 4; LDLR, low-density lipoprotein receptor; ICAM-1, intercellular adhesion molecule–1; NCL, nucleolin; HSPG, the gene for perlecan. (**C**) NAECs from wheeze groups have decreased expression of MX1, a marker of cellular antiviral response. (**D**) NAECs from wheeze groups have decreased expression of IFNAR1, another antiviral factor. (**E**) NAECs were differentiated at ALI. TEER permeability was determined at various days post-ALI. *n* = 5 to 6 in each group and data is shown as mean (SD). *P < 0.05, two-way analysis of variance (ANOVA) with repeated measures and Tukey post hoc test. (**F** to **H**) After fully differentiated, NAECs were infected with RSV 01/2-20 (MOI = 3). Twenty-four hours postinfection, cells were harvested and *RSV M* gene expression (F) or IFNL2 (G) was determined by quantitative polymerase chain reaction (qPCR) and normalized to TATA box–binding protein 1 (TBP1). IFN-β protein levels were measured by enzyme-linked immunosorbent assay (ELISA) in basolateral supernatants of ALI cultures (H). *n* = 5 to 6 samples from different participants in each group, and data are shown as mean (SD). *P < 0.05 and **P < 0.01, two-way ANOVA with Tukey post hoc test (E) and one-way ANOVA with Kruskal-Wallis post hoc test [(F) to (H)]. n.s., not significant.

NAECs from wheeze groups showed increased diversity and altered expression profile of currently known RSV receptors across different epithelial subsets in RSV and wheeze subgroups (Fig. 5, A and B). RSV receptor genes for this panel were determined on the basis of previously published reports (*19*, *20*). Moreover, NAECs from wheeze groups had decreased expression of *MX1* (MX dynamin-like guanosine triphosphatase 1) and *IFNAR1* (interferon-α/β receptor α chain), key markers of cellular antiviral responses (Fig. 5, C and D). On the basis of these data, we wanted to explore whether differentiated NAECs had different RSV infectivity in culture. To do this, NAECs from five to six unique children in each of the four a priori selected groups [no wheeze/no RSV (control), wheeze/no RSV, wheeze/RSV, and no wheeze/RSV] were differentiated. Starting at the time of ALI (day 7), TEER permeability was assessed as a measure of barrier capacity of the NAEC cultures from the four groups. TEER permeability measurements were reduced in NAECs from wheeze and wheeze/RSV children compared to NAECs from control or no wheeze/RSV children (Fig. 5E). At day 21 of ALI, after full differentiation, NAECs were infected in vitro with a clinically relevant strain of RSV (RSV 01/2-20) at a multiplicity of infection (MOI) = 3 or mock infection. Expression of *RSV M* gene was determined 24 hours postinfection, and NAECs from wheeze/RSV children had increased *RSV M* gene expression compared to NAECs from control and no wheeze/RSV children (Fig. 5F). IFN production is known to increase after RSV infection as an initial antiviral response. Therefore, we also measured IFN-λ (*IFNL2*) gene expression and IFN-β protein levels. NAECs from control (no wheeze/no infant RSV infection) and RSV (no wheeze/RSV) children had increased *IFNL2* gene and IFN-β protein expression after in vitro RSV 01-2/20 infection compared to NAECs from children with wheeze/no RSV infection and wheeze/RSV phenotypes (Fig. 5, G and H). Together, these findings suggest that wheeze is an underlying phenotype with potentially genetic or epigenetic origins and is characterized by increased barrier permeability and diversity of RSV receptors, which may predispose to infection, enhance RSV infection severity in infancy, or lead long-term effects of RSV infection.

## DISCUSSION

Using NAECs from children who had careful characterization of RSV infection and wheeze in the first year of life and single-cell profiling of these cells collected at the end point of postnatal differentiation (age 2 to 3 years), we demonstrated that NAECs from children with a wheeze phenotype are characterized by an altered ALI differentiation trajectory in culture with early basal activation of developmental pathways, plasticity in precursor differentiation, and persistent expansion of developmentally active epithelial cell subsets. Our findings of differentiation defects in ex vivo cultures point to changes in developmental pathways and a true developmentally reprogrammed phenotype resulting in observed epithelial culture growth alterations in 2- to 3-year-old children who develop wheeze. NAECs from children with wheeze also have increased diversity of all currently known RSV receptors and blunted antiviral immune responses to in vitro infection. Together, our findings suggest that airway epithelial developmental reprogramming in children with wheeze has a genetic or epigenetic origin, which is characterized by increased barrier permeability and altered antiviral response in addition to increased diversity of RSV receptors that may predispose to and amplify the effects of RSV infection in infancy. Early-life RSV infection results in an additive effect on aberrant airway epithelial differentiation most pronounced in those with a wheeze phenotype, suggesting a potential gene by environment interaction that may contribute to and explain the association of early-life RSV infection with childhood asthma risk.

One of the remarkable findings in our study was the level of epithelial developmental reprogramming associated with an early-life wheeze phenotype compared with nonwheezers. AECs from children with an early-life wheeze phenotype (wheeze with or without RSV) also had more pronounced epithelial reprogramming than cells from children that had RSV in infancy without wheeze. Our findings point to developmental defects originating in basal cells of children with wheeze, which include increased expression of developmental pathways (WNT, Notch/Jagged, TGFβ, and EGF), tissue plasminogen system activation, and increased production of extracellular matrix proteins (versican, fibronectin, periostin, tenascin C, among others). Markers such as periostin and PAI-2 also traditionally serve as markers of “type 2–high” molecular endotype of allergic asthma. The early appearance and persistence of developmental defects in these pathways in epithelial culture among children with a wheeze phenotype may suggest developmental/epigenetic origins of asthma that might initiate persistent airway inflammation in some children and promote the development of allergic inflammation. In support of this concept, we and others have reported dysregulation of epithelial developmental pathways in wheeze and adult asthma (*21*–*24*).

Nasal airway epithelial culture in ALI allowed for the in vitro assessment of epithelial developmental processes from basal to specialized differentiated cells. If terminology of heterochrony (study of differences in the timing, rate, or duration of a developmental process) were to be used, then the effect of basal reprogramming on the wheeze NAEC differentiation could be characterized as an “early developmental onset–delayed developmental offset” hypermorphosis (“hyperdevelopmental” trajectory) (*25*). Our lineage reconstruction analysis demonstrated persistence and proliferation of specific epithelial cell subsets including early progenitor and club precursor epithelial cells. Our investigation of club precursor cells from children with a wheeze phenotype determined that they express KRT13, which, in conjunction with club precursor markers, would potentially classify them as recently found “hillock” cells (*26*). However, little is currently known about the role and identity of hillock cells, especially in wheezing illnesses and asthma. Montoro *et al.* (*26*) described them as a variety of club cells descending from the basal cell lineage and expressing keratin 13. These cells are found in contiguous groups of stratified epithelial cells, forming structures termed “hillocks,” where they have high cycling capacity and express markers associated with squamous epithelial differentiation, cellular adhesion, and immunomodulation (*26*, *27*). These cells were also recently reported in a human model of asthma exacerbation in vitro associated with an allergic asthma phenotype (*28*). Since this transient population between basal and club cells was associated with abnormal epithelial differentiation, we can deduce that this reprogrammed epithelial subset is playing a role in perpetual epithelial barrier injury-repair/remodeling processes (or a transient role in normal wound healing). Our findings suggest that transient phenotypes are part of a broader developmental reprogramming following preschool wheezing illnesses and could be markers of abnormal barrier development in early life. Moreover, abnormal wheeze differentiation was associated with persistence of complement system and immune gene expression (i.e., *IL32, CXCL3, CXCL8, HLA-DRA, HLA-DRB1, HLA-DQA1*, and *CFB*) in different epithelial subsets (parabasal, early progenitor, and goblet). This may indicate a baseline inflammatory state of the epithelium even in the absence of active interaction with immune cells (*29*, *30*) and may lead to abnormal immune responses at the epithelial barrier interface.

The impact of RSV on in vivo differentiation of epithelium early in life and the development of asthma had been unexplored. RSV can reprogram differentiation of AECs in vitro by infecting and altering the developmental course of epithelial progenitor cells (*31*). However, our study uncovered the significance of a prewheeze epithelial developmental phenotype as a potential predisposition factor for aberrant barrier function, enhanced risk for RSV infection, and perhaps infection severity. Despite these important observations, whether RSV drives wheeze or uncovers a predisposition to it remains a major controversy in the field, which we anticipate future longitudinal studies that include airway epithelial samples beginning at birth will be able to resolve. While we describe broader changes in cell types and pathways associated with the wheeze phenotype (basal subset activation and mucociliary precursor expansion) than with infant RSV infection, it is also important to note that if we assessed gene expression during active RSV infection (as opposed to the reprogramming effect due to earlier RSV exposures in infancy), then more gene expression might have been expected. In combination, there appears to be an additive effect of RSV and wheeze on aberrant airway epithelial development. It is critical to determine which factors drive this early susceptibility. Underlying predisposition of wheeze phenotype to RSV infection is likely due to genetic or epigenetic developmental reprogramming (which includes decrease in antiviral defense capacity) due to a combination of genetic and environmental factors, which potentially represents a “second hit” to increase the impact of RSV infection on the epithelium. One intriguing possibility is reprogramming associated with change in systemic and cellular metabolism, which is a known driver of developmental epigenetic change. We also found altered expression of RSV receptors on epithelial cells of children with an early wheeze phenotype. Some of the suspected RSV-associated receptors [IGF1R, heparan sulfate proteoglycan, and EGF receptor (EGFR)] are also active participants in epithelial and matrix remodeling, which suggests that barrier remodeling processes that we identified as part of the wheeze phenotype may drive increased receptor expression for RSV. Moreover, we found down-regulation of antiviral response markers, such as MX1 and IFNAR1, in the epithelial cells from children with wheeze, which suggest that developmental reprogramming in wheeze affects the ability of the airway epithelium to mount antiviral defense. This finding in epithelium is consistent with our finding of suppressed antiviral immunity in peripheral blood mononuclear cells from children with prior RSV infection in infancy (*32*) and previous reports in childhood allergic asthma (*33*). The functional consequences of this are supported by our findings of increased in vitro RSV infection as measured by *RSV M* gene expression and decreased expression of *IFNL2* in NAECs from children with an early-life wheeze phenotype compared to NAECs from nonwheezers. Further, children with both an early-life wheeze phenotype and infant RSV infection had the most marked changes in epithelial differentiation and function relative to controls. The distinct differences in pathways associated with the wheeze phenotype and early-life RSV infection may suggest a “double hit” scenario in epithelial development in infancy: an early wheeze phenotype at or after birth due to genetic or epigenetic modification with increased susceptibility to RSV and early-life infection further amplifying developmental defects in early life and leading to differentiation to a functionally impaired airway barrier. This also supports a shared genetic predisposition for both wheeze/asthma and more severe RSV infection that we have previously demonstrated (*34*). An airway developmental trajectory characterized by barrier disruption and enhanced susceptibility to infection may predispose a child to increased immune mucosal surveillance, higher penetrance, and processing of environmental antigens, leading to atopy and allergic disease, as well as enhanced susceptibility to asthma risk factors such as secondhand smoke and air pollution.

This study uses primary NAECs collected from children at age 2 to 3, the end point of postnatal epithelial differentiation, who also had careful characterization of both RSV infection and wheeze in early life. Limitations of the study include cross-sectional collection of AECs for the main analysis. We performed scRNA-seq analysis of 2- to 3-year-old NAEC differentiation in ALI culture, which captured the basal-to-mature redifferentiation process rather than a childhood epithelial developmental curve or in situ differences between established airway subsets. Small sample size is another limitation, which limited power to observe DEGs. While epithelial cell populations functionally differ between the upper and lower airway, basal–to–specialized epithelial differentiation pathways are remarkably similar in terms of developmental processes in the upper and lower airway, and transcriptomic data support important parallels between the upper and lower airway epithelium. This supports the use of NAECs in children to model the differences in airway epithelial development that characterize wheeze and respiratory viral infection (*13*, *14*, *35*).

This study provides evidence for how the airway epithelium develops in disease and how and why it responds differently to environmental stress. Understanding development in the airway epithelial barrier after birth through early childhood is recognized as a key to understanding the developmental origins of childhood wheeze and asthma. The airway epithelium is a fundamental mucosal barrier, and the nasal epithelium provides critical first responses against viral infections and illnesses. The findings of our study suggest that RSV infection and wheezing illnesses alter nasal airway epithelial development including barrier function and the response to respiratory viruses. We postulate that wheezing illnesses and early-life RSV infection might change in the airway epithelium in some children to enhance susceptibility to respiratory viral infections, thus increasing the risk of additional wheezing illnesses and possible chronic airway obstruction. As there are no effective primary preventive interventions for asthma, identifying the timing and pathways driving airway epithelial development may inform additional targets for prevention and treatment approaches that regulate the normal development of the early-life airway epithelium with the potential to prevent both severe respiratory viral infections and asthma.

## MATERIALS AND METHODS

### Study population

The INSPIRE is a large, population-based birth cohort of healthy, term children (*n* = 1946) specifically designed to test the association of RSV infection in infancy with risk of childhood asthma. RSV infection status (uninfected versus infected) was ascertained in the first year of life using a combination of passive and active surveillance with viral identification through molecular and serological testing. Children were followed prospectively for annual recurrent wheeze using a validated questionnaire (*16*, *17*, *36*). The study population in this proposal is an a priori–designed nested cohort of 100 participants selected for follow-up using a random number generator from four groups of children with and without wheezing and RSV-infected and uninfected during infancy [no wheeze/no RSV (controls), no wheeze/RSV, wheeze/RSV, and wheeze/no RSV] (fig. S1). These children completed additional in-person study visits between ages 2 and 3 years that included NAEC collection and culture representing the end point of postnatal airway epithelial differentiation. From this nested cohort of 100 children, two to three participants in each of the four groups were randomly selected. We selected samples from children with wheeze who had wheeze during follow-up between ages 1 and 4. All NAECs were differentiated in ALI culture and sent for scRNA-seq. In additional experiments, five to six individual participants were selected from each of the four groups, and NAECs were differentiated and TEER was measured during differentiation. These NAECs were infected with RSV 01/2-20 or mock for follow-up studies. The Institutional Review Board of Vanderbilt University Medical Center approved this study and one parent of each child provided informed consent for their participation. The detailed methods for INSPIRE have been previously reported (*36*).

### Human NAEC culture

NAECs were collected from the a priori nested cohort of INSPIRE children during a well-child visit between 2 and 3 years of age. Children were screened for signs and symptoms of respiratory illness, and, if detected, visits and collections were rescheduled. NAECs were collected from individuals by brushing nasal passages at the level of the inferior turbinate with a soft flocked cotton swab (Copan, Murrieta, CA, USA) and placing them in cold PneumaCult-Ex Plus Medium (STEMCELL Technologies, Vancouver, Canada). Cells were placed on collagen-coated flasks and then were submerged and expanded in PneumaCult-Ex Plus Medium, consisting of 500 ml of Ex Plus Basal Medium supplemented with 10 ml of PneumaCult-Ex Plus 50× supplement, 0.5 ml of hydrocortisone stock solution, and penicillin-streptomycin (Pen-Strep). Once cells reached 50 to 70% confluence on the flask, NAECs were disassociated using 2 to 4 ml of animal component–free (ACF) cell dissociation solution and 2 to 4 ml of ACF inhibition solution (STEMCELL Technologies, Vancouver, Canada) and transferred to a 24-transwell plate with transwells coated in 200 μl of rat tail collagen I. Cells remained submerged in PneumaCult-Ex Plus Medium until confluency (~1 week) with medium changed every other day. Once confluent, medium was removed from the apical chamber, and the NAECs were allowed to differentiate for 3 weeks using PneumaCult-ALI Medium (500 μl) in the basal chamber. Medium in the basal chamber was changed every other day. Complete PneumaCult-ALI Medium consists of 490 ml of ALI Medium plus 50 ml of PneumaCult-ALI 10× supplement, 5 ml of PneumaCult-ALI maintenance supplements, 2.5 ml of hydrocortisone stock solution, and 1% Pen-Strep. Laboratory staff involved in NAEC culture, TEER measurements, and in vitro RSV infection were blinded to group assignment.

### Single-cell RNA sequencing

For each sample sequenced (*n* = 2 to 3 per group), 10,000 cell target was used for single-cell capturing and library construction using Chromium Single-Cell 3’ Reagent Kits (v3.1 chemistry, PN-1000130) from 10x Genomics, according to the manufacturer’s instructions. Single-cell Gel Beads-in-Emulsion–captured cells underwent lysis and transcript barcoding. The corresponding cDNA along with cell barcodes were amplified using polymerase chain reaction (PCR). scRNA-seq libraries were constructed using 10x Genomics Library Construction kits (PN-1000196) and Dual Index Kit TT Set A (PN-1000215). The constructed libraries were sequenced on an Illumina platform to generate paired end reads. The resulting raw sequencing data were processed using the CellRanger pipeline (version 7.1.0, 10x genomics).

### In vitro RSV infection of NAECs

After NAECs were fully differentiated (>21 days at ALI), each of the donor cells were infected on the apical surface (top chamber) with 50 μl of RSV 01/2-20 (MOI = 3) or mock. After infection, cells were incubated for an hour at 37°C with gentle rocking. After an hour, inoculum was removed from the apical surface, cells were washed 1× with phosphate-buffered saline (PBS), and cells were placed back into the appropriate temperature incubator. Basolateral supernatant, viral washing, and cells were collected at 24 hours postinfection.

### TEER measurements

The barrier function of NAECs was determined by measuring the TEER as previously described (*37*) at days 7, 14, and 21 of ALI differentiation. Cells were allowed to equilibrate to room temperature for 15 min before TEER measurement. ALI medium was replaced by PBS on the basolateral side, and PBS was added to the apical side of each transwell before TEER measurement. TEER was measured under a sterile hood with a chopstick electrode and an epithelial voltammeter (EVOM^2^) (World Precision Instruments, Sarasota, FL). Each well was measured in triplicate, and values were averaged. After measurement, PBS was removed, and ALI medium was replenished on the basolateral side.

### RNA isolation, cDNA generation, and quantitative PCR

For RNA isolation, lysed cells were thawed then passed through a QIAshredder (QIAGEN, Hilden, Germany). RNA was then extracted using the RNeasy Mini Kit (QIAGEN, Hilden, Germany). RNA quality was assessed using a NanoDrop 2000 spectrophotometer (Thermo Fisher Scientific, Waltham, USA). cDNA was generated using the SuperScript IV First-Strand Synthesis Kit (Thermo Fisher Scientific, Waltham, USA). Quantitative PCR (qPCR) was conducted using QuantStudio, and gene expression was normalized to a housekeeping gene of TATA box–binding protein 1 (TBP1). RNA M gene primers (forward, GGC AAA TAT GGA AAC ATA CGT GAA; reverse, TCT TTT TCT AGG ACA TTG TAY TGA ACA) were generated using IDT. IFNL2 gene primer (catalog number: 4331182, assay ID: Hs00820125_g1) and housekeeping gene TBP primer (catalog number: 4331182, assay ID: Hs00427620) were purchased from Thermo Fischer Scientific (Waltham, MA, USA).

### IFN-β enzyme-linked immunosorbent assay

Basolateral supernatants of ALI cultures were used to measure IFN-b via a Quantikine enzyme-linked immunosorbent assay (ELISA) (R&D Systems, catalog number: QK410) per the manufacturer’s instructions. Any value below the limit of detection was assigned half the value of the lowest detectable standard.

### Bioinformatic and statistical analysis

Seurat R package version 4.1.1 was used for analyzing counts data, normalization, dimension reduction, clustering, integration, visualization, identification of unique cluster markers, and differential gene expression analysis. We filtered out the cells that have unique feature counts over 9500 or less than 200 based on the pattern of the dataset. We also filtered out cells with mitochondrial counts of >5%. Seurat harmony integration was performed on the Seurat object, correcting for batch and sample variability. For additional validation, the dataset was split by group and reintegrated to check for consistency in clustering. The integrated dataset was stable after reintegration. Cell cycle analysis was performed using cc.genes function in Seurat. Slingshot v.2.2.1.R package was used for differentiation trajectory inference. Ggplot2 R package version 3.4.0 was used for figure generation and visualization. DEGs were determined using nonparametric Wilcoxon rank sum test implemented in Seurat. In addition, pseudobulking analysis of the basal cell cluster was performed by sample, and DEGs were assessed via DESeq2 implemented in Seurat (see datasets S1 to S3 for pseudobulk analysis output). EnhancedVolcano R package was used to generate volcano plots in Fig. 4C based on Seurat DEG analysis. The data mining results shown in Fig. 3A are based on human lung RNA-seq data (neonate, infant, and child) generated by the LungMAP Consortium and downloaded from (www.lungmap.net) in 2020. The LungMAP consortium and the LungMAP Data Coordinating Center (U24-HL148865) are funded by the National Heart, Lung, and Blood Institute (*38*). Pathway analysis in Fig. 4D was performed in Metaboanalyst (*39*) (“genes only” option) using Kyoto Encyclopedia of Genes and Genomes (KEGG) pathway reference and hypergeometric test–based enrichment analysis. We obtained biological processes in fig. S2A associated with the genes identified from our differential expression analysis using Enrichr (*40*) with Reactome and KEGG-based pathway analysis with hypergeometric statistical testing and Clustergrammer hierarchical clustering. TEER in Fig. 5E was analyzed using two-way analysis of variance (ANOVA) with repeated measures and a Tukey post hoc test and presented as a line graph depicting the means ± SD with colors of groups shown in the legend. The data in Fig. 5 (F to H) were analyzed using one-way ANOVA with a Kruskal-Wallis post hoc test and presented as bar graphs depicting the means ± SD with sample number listed in each figure legend.

## Supplementary Materials

### The PDF file includes:

Tables S1 and S2 Figs. S1 and S2 Legends for datasets S1 to S3

### Other Supplementary Material for this manuscript includes the following:

Datasets S1 to S3
