# Supplementary material for: Single-cell profiling demonstrates the combined effect of wheeze phenotype and infant viral infection on airway epithelial development
Source: Sci Adv. 2025 May 23;11(21):eadr9995. doi: 10.1126/sciadv.adr9995 (PMC12101503; doi:10.1126/sciadv.adr9995)
Supplement: Supplementary file 1 — Tables S1 and S2 Figs. S1 and S2 Legends for datasets S1 to S3 [file sciadv.adr9995_sm.pdf]

Science Advances
AAAS

Supplementary Materials for

Single-cell profiling demonstrates the combined effect of wheeze phenotype and infant viral infection on airway epithelial development

Sergejs Berdnikovs *et al.*

Corresponding author: Email: Sergejs Berdnikovs, sergejs.berdnikovs@cuanschutz.edu;
Dawn C. Newcomb, dawn.newcomb@vumc.org

Sci. Adv. **11**, eadr9995 (2025)
DOI: 10.1126/sciadv.adr9995

The PDF file includes:

Tables S1 and S2
Figs. S1 and S2
Legends for datasets S1 to S3

Other Supplementary Material for this manuscript includes the following:

Datasets S1 to S3

Supplementary Table 1. Baseline demographic characteristics and outcomes of study participants selected for this nested cohort study (N=9), the cohort excluding the 9 nested cohort participants and the entire cohort from which they were selected. The bottom section of the table includes the child clinical outcomes at age 3-4 years.

	**Participants included in nested cohort N=9**	**Full cohort excluding 9 participants in nested cohort**	**Full cohort N=1946**
**Baseline demographic characteristics captured near birth**			
RSV season			
2012-13		44%	44%
2013-14	100%*	56%	56%
Sex			
Female	11%	48%	48%
Male	89%	52%	52%
Birth weight, grams (median, IQR)	3547 (3121, 3641)	3405 (3120, 3745)	3405 (3120, 3740)
Gestational age, weeks (median, IQR)	39 (38, 39)	39 (39-40)	39 (39-40)
Race			
Black, non-Hispanic	44%	18%	18%
White, non-Hispanic	44%	65%	65%
Hispanic	0%	9%	9%
Other (multi-race or unknown)	11%	9%	9%
Secondhand smoke Exposure (maternal smoking during pregnancy and/or infancy)	0%	22%	22%
Maternal atopy	33%	41%	41%
Maternal asthma	11%	20%	19%
**Child clinical outcomes following inclusion in nested cohort study**			
Aeroallergen sensitization at 3 years	50%	44% (with follow up at 4 years N=1240)	44% (with follow up at 4 years N=1248)
Asthma at 4 years	33%	19% (with follow up at 4 years N=1486)	19% (with follow up at 4 years N=1495)

* Subjects all selected from a single year by design, as it is only enrolled children from this birth cohort year that were included.

Supplementary Table 2. Demographic characteristics of study participants selected for this nested cohort study (N=9) stratified by the *a priori* selected four study groups based on RSV infection ((+) indicates infection in the first year of life, (-) indicates no infection in the first year of life based on nasal PCR and serology) and presence of wheeze at age 1 year ((+) indicates wheeze in the first year of life, (-) indicates no wheeze in the first year of life based validated surveys).

	**RSV (–) Wheeze(–) (N = 2)**	**RSV (–) Wheeze (+) (N = 2)**	**RSV (+) Wheeze (–) (N = 3)**	**RSV (+) Wheeze (+) (N = 2)**	**Combined study group**
**Characteristics of infants captured at enrollment near birth**					
Sex<br>Female<br>Male	0%<br>100%	0%<br>100%	0%<br>100%	50%<br>50%	11%<br>89%
Maternal atopy	0%	50%	33%	50%	33%
**Outcomes 2-3 years following selection into sub-study**					
3-year aeroallergen sensitization	100%	50%	0%	100%	50%
4-year asthma	0%	100%	0%	50%	33%

Fig. S1

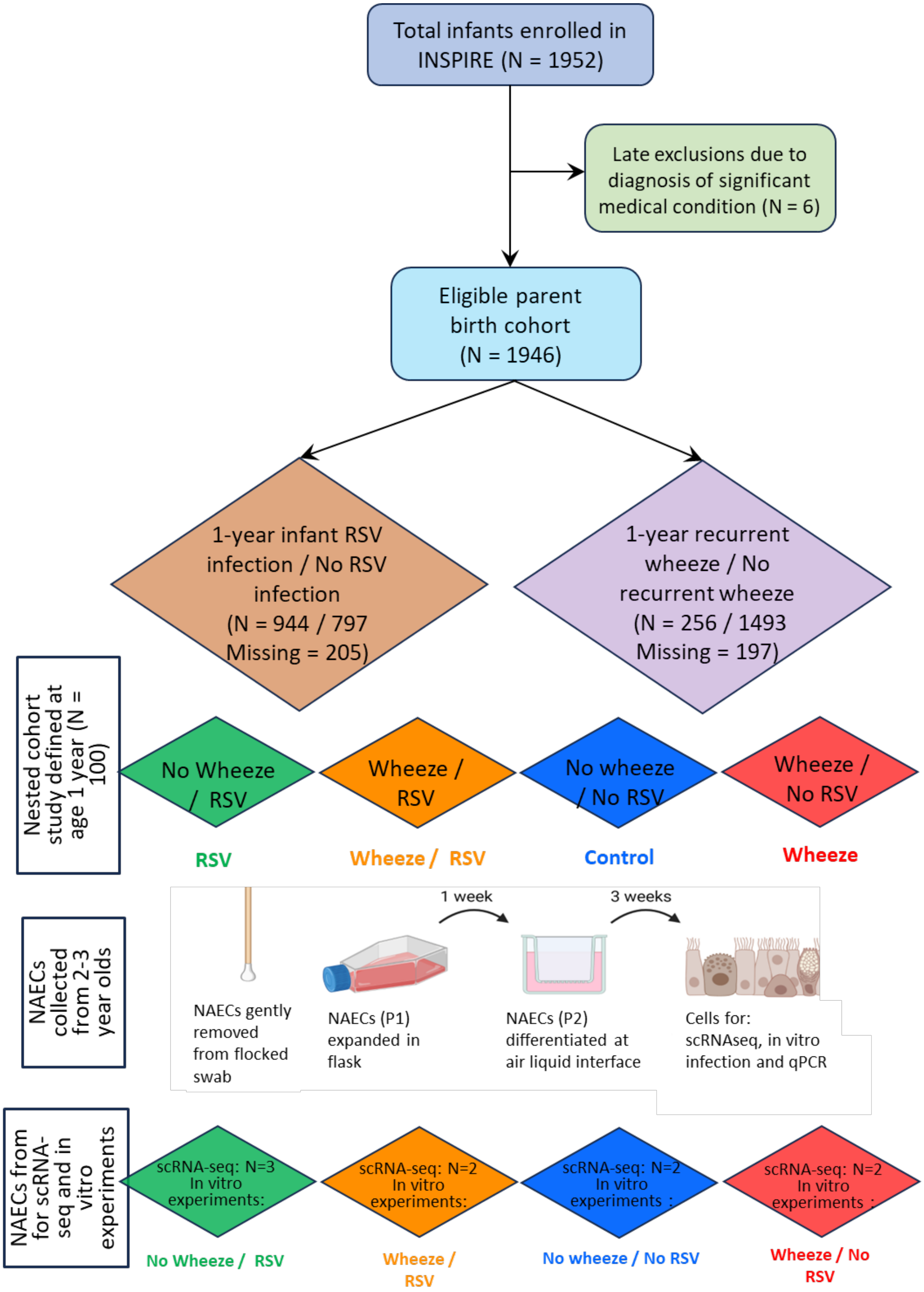

Total infants enrolled in INSPIRE (N = 1952)
Late exclusions due to diagnosis of significant medical condition (N = 6)
Eligible parent birth cohort (N = 1946)
1-year infant RSV infection / No RSV infection (N = 944 / 797 Missing = 205)
1-year recurrent wheeze / No recurrent wheeze (N = 256 / 1493 Missing = 197)
Nested cohort study defined at age 1 year (N = 100)
No Wheeze / RSV
Wheeze / RSV
No wheeze / No RSV
Wheeze / No RSV
RSV
Wheeze / RSV
Control
Wheeze
NAECs collected from 2-3 year olds
1 week
3 weeks
NAECs gently removed from flocked swab
NAECs (P1) expanded in flask
NAECs (P2) differentiated at air liquid interface
Cells for: scRNAseq, in vitro infection and qPCR
NAECs from for scRNA-seq and in vitro experiments
scRNA-seq: N=3 In vitro experiments:
scRNA-seq: N=2 In vitro experiments:
scRNA-seq: N=2 In vitro experiments :
scRNA-seq: N=2 In vitro experiments :
No Wheeze / RSV
Wheeze / RSV
No wheeze / No RSV
Wheeze / No RSV

Fig S1. Flow diagram of *a priori* designed nested cohort study of 100 INSPIRE participants selected into one of four mutually exclusive groups with recurrent wheeze/no recurrent wheeze during the first year of life using validated questionnaires, and RSV infection/no RSV infection during the first year of life as determined by active surveillance using PCR and 1-year RSV serology. These 100 children were seen for an additional research visit at age 2.5 years with collection of nasal airway epithelial cells (NAEC) for culture and RNA-sequencing. A randomly selected group of children with NAEC in each of the four groups were randomly selected for single cell RNA-sequencing, and for culture in air liquid interface for *in vitro* infection with clinical isolates of RSV to measure RSV infectivity and barrier permeability using transepithelial electrical resistance (TEER). All male samples were selected unless there were no available samples.

Fig. S2

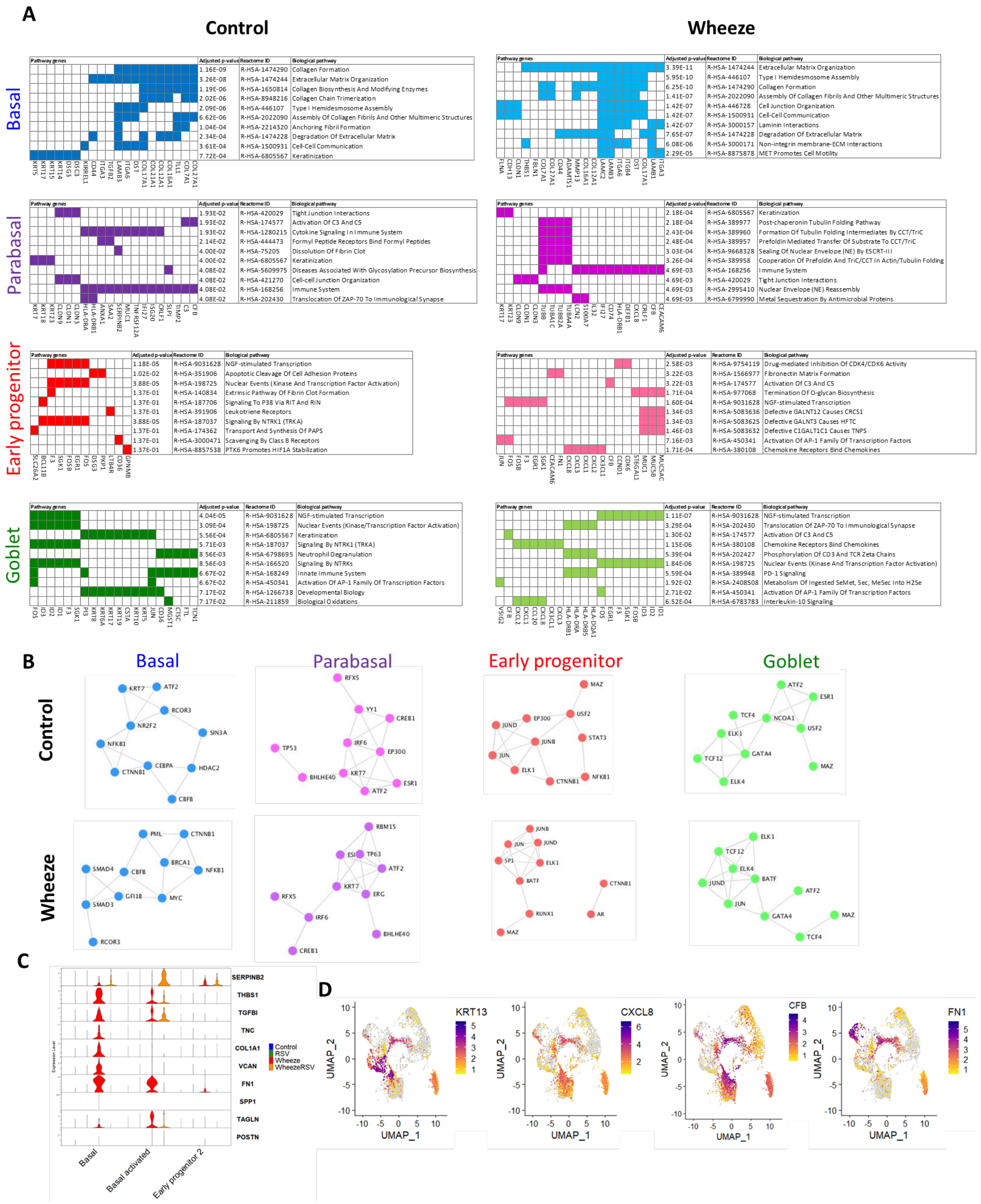
A
Control
Wheeze
Basal
Parabasal
Early progenitor
Goblet
Pathway genes
Adjusted p-value
Reactome ID
Biological pathway
1.16E-09 R-HSA-1474290 Collagen Formation
3.26E-08 R-HSA-1474244 Extracellular Matrix Organization
1.19E-06 R-HSA-1650814 Collagen Biosynthesis And Modifying Enzymes
2.02E-06 R-HSA-8948216 Collagen Chain Trimerization
2.09E-06 R-HSA-446107 Type I Hemidesmosome Assembly
6.62E-06 R-HSA-2022090 Assembly Of Collagen Fibrils And Other Multimeric Structures
1.04E-04 R-HSA-2214320 Anchoring Fibril Formation
2.34E-04 R-HSA-1474228 Degradation Of Extracellular Matrix
3.61E-04 R-HSA-1500931 Cell-Cell Communication
7.72E-04 R-HSA-6805567 Keratinization
3.39E-11 R-HSA-1474244 Extracellular Matrix Organization
5.95E-10 R-HSA-446107 Type I Hemidesmosome Assembly
6.25E-10 R-HSA-1474290 Collagen Formation
1.41E-07 R-HSA-2022090 Assembly Of Collagen Fibrils And Other Multimeric Structures
1.42E-07 R-HSA-446728 Cell Junction Organization
1.42E-07 R-HSA-1500931 Cell-Cell Communication
1.42E-07 R-HSA-3000157 Laminin Interactions
7.65E-07 R-HSA-1474228 Degradation Of Extracellular Matrix
6.08E-06 R-HSA-3000171 Non-integrin membrane-ECM Interactions
2.29E-05 R-HSA-8875878 MET Promotes Cell Motility
1.93E-02 R-HSA-420029 Tight Junction Interactions
1.93E-02 R-HSA-174577 Activation Of C3 And C5
1.93E-02 R-HSA-1280215 Cytokine Signaling In Immune System
2.14E-02 R-HSA-444473 Formyl Peptide Receptors Bind Formyl Peptides
4.00E-02 R-HSA-75205 Dissolution Of Fibrin Clot
4.00E-02 R-HSA-6805567 Keratinization
4.08E-02 R-HSA-5609975 Diseases Associated With Glycosylation Precursor Biosynthesis
4.08E-02 R-HSA-421270 Cell-cell Junction Organization
4.08E-02 R-HSA-168256 Immune System
4.08E-02 R-HSA-202430 Translocation Of ZAP-70 To Immunological Synapse
2.18E-04 R-HSA-6805567 Keratinization
2.18E-04 R-HSA-389977 Post-chaperonin Tubulin Folding Pathway
2.43E-04 R-HSA-389960 Formation Of Tubulin Folding Intermediates By CCT/TriC
2.48E-04 R-HSA-389957 Prefoldin Mediated Transfer Of Substrate To CCT/TriC
3.03E-04 R-HSA-9668328 Sealing Of Nuclear Envelope (NE) By ESCRT-III
3.26E-04 R-HSA-389958 Cooperation Of Prefoldin And TriC/CCT In Actin/Tubulin Folding
4.69E-03 R-HSA-168256 Immune System
4.69E-03 R-HSA-420029 Tight Junction Interactions
4.69E-03 R-HSA-2995410 Nuclear Envelope (NE) Reassembly
4.69E-03 R-HSA-6799990 Metal Sequestration By Antimicrobial Proteins
1.18E-05 R-HSA-9031628 NGF-stimulated Transcription
1.02E-02 R-HSA-351906 Apoptotic Cleavage Of Cell Adhesion Proteins
3.88E-05 R-HSA-198725 Nuclear Events (Kinase And Transcription Factor Activation)
1.37E-01 R-HSA-140834 Extrinsic Pathway Of Fibrin Clot Formation
1.37E-01 R-HSA-187706 Signaling To P38 Via RIT And RIN
1.37E-01 R-HSA-391906 Leukotriene Receptors
3.88E-05 R-HSA-187037 Signaling By NTRK1 (TRKA)
1.37E-01 R-HSA-174362 Transport And Synthesis Of PAPS
1.37E-01 R-HSA-3000471 Scavenging By Class B Receptors
1.37E-01 R-HSA-8857538 PTK6 Promotes HIF1A Stabilization
2.58E-03 R-HSA-9754119 Drug-mediated Inhibition Of CDK4/CDK6 Activity
3.22E-03 R-HSA-1566977 Fibronectin Matrix Formation
3.22E-03 R-HSA-174577 Activation Of C3 And C5
1.71E-04 R-HSA-977068 Termination Of O-glycan Biosynthesis
1.60E-04 R-HSA-9031628 NGF-stimulated Transcription
1.34E-03 R-HSA-5083636 Defective GALNT12 Causes CRCS1
1.34E-03 R-HSA-5083625 Defective GALNT3 Causes HFTC
1.46E-03 R-HSA-5083632 Defective C1GALT1C1 Causes TNPS
7.16E-03 R-HSA-450341 Activation Of AP-1 Family Of Transcription Factors
1.71E-04 R-HSA-380108 Chemokine Receptors Bind Chemokines
4.04E-05 R-HSA-9031628 NGF-stimulated Transcription
3.09E-04 R-HSA-198725 Nuclear Events (Kinase/Transcription Factor Activation)
5.56E-04 R-HSA-6805567 Keratinization
5.71E-03 R-HSA-187037 Signaling By NTRK1 (TRKA)
8.56E-03 R-HSA-6798695 Neutrophil Degranulation
8.56E-03 R-HSA-166520 Signaling By NTRKs
6.67E-02 R-HSA-168249 Innate Immune System
6.67E-02 R-HSA-450341 Activation Of AP-1 Family Of Transcription Factors
7.17E-02 R-HSA-1266738 Developmental Biology
7.17E-02 R-HSA-211859 Biological Oxidations
1.11E-07 R-HSA-9031628 NGF-stimulated Transcription
3.29E-04 R-HSA-202430 Translocation Of ZAP-70 To Immunological Synapse
1.30E-02 R-HSA-174577 Activation Of C3 And C5
1.15E-06 R-HSA-380108 Chemokine Receptors Bind Chemokines
5.39E-04 R-HSA-202427 Phosphorylation Of CD3 And TCR Zeta Chains
1.84E-06 R-HSA-198725 Nuclear Events (Kinase And Transcription Factor Activation)
5.59E-04 R-HSA-389948 PD-1 Signaling
1.92E-02 R-HSA-2408508 Metabolism Of Ingested SeMet, Sec, MeSec Into H2Se
2.71E-02 R-HSA-450341 Activation Of AP-1 Family Of Transcription Factors
6.52E-04 R-HSA-6783783 Interleukin-10 Signaling
B
Basal
Parabasal
Early progenitor
Goblet
Control
Wheeze
C
SERPINB2
THBS1
TGFBI
TNC
COL1A1
VCAN
FN1
SPP1
TAGLN
POSTN
Expression Level
Control
RSV
Wheeze
WheezeRSV
Basal
Basal activated
Early progenitor 2
D
KRT13
CXCL8
CFB
FN1
UMAP_1
UMAP_2

Fig. S2. NAECs from wheeze study groups show abnormal and sustained biological processes associated with extracellular matrix deposition and immune activation. A. Biological enrichment analysis of genes expressed in different developmental clusters by study group. **B.** Transcriptional factor inference. **C.** Expression of gene markers associated with remodeling, immune and tissue plasminogen system activation in control and wheeze groups. **D.** Expression of KRT13 (epithelial marker), CXCL8 (immune marker), CFB (complement) and FN1 (fibronectin) in wheeze epithelial cells.

Supplementary Excel Dataset 1. RSV vs Control pseudobulk analysis output for basal cell cluster differential gene expression.

Supplementary Excel Dataset 2. Wheeze vs Control pseudobulk analysis output for basal cell cluster differential gene expression.

Supplementary Excel Dataset 3. WheezeRSV vs Control pseudobulk analysis output for basal cell cluster differential gene expression.
